# Flame-Retardant Foamed Material Based on Modified Corn Straw Using Two Nitrogenous Layers

**DOI:** 10.3390/ma16030952

**Published:** 2023-01-19

**Authors:** Qiong Su, Hongling Wang, Yanbin Wang, Shuang Liang, Shaofeng Pang, Xiangfei Zhao, Xiyang Sun, Xiaoqin Shi, Jun Zhao

**Affiliations:** 1 School of Chemical Engineering, Northwest Minzu University, Lanzhou 730030, China; 2 Key Laboratory of Environment-Friendly Composite Materials of the State Ethnic Affairs Commission, Lanzhou 730030, China; 3 Engineering Research Center of Biomass-Functional Composite Materials of Gansu Province, Lanzhou 730030, China; 4 Key Laboratory of Utility of Environmental Friendly Composite Materials and Biomass in Universities of Gansu Province, Lanzhou 730030, China

**Keywords:** foamed material, corn straw, flame-retardant, nitrogenous layers, graft, ionic liquid

## Abstract

Foamed materials based on a biopolymer of crop straws are environmentally friendly, but ignitability limits their application. In this study, two nitrogenous layers were introduced onto corn straw by esterification and grafting for flame-retardant purposes. The inner thin nitrogenous layer consisted of imidazole rings, and the outer thick nitrogenous layer consisted of grafted acrylamide by a free-radical polymerization. The outer nitrogenous layer was simultaneously introduced into the system with a foaming process at 150 °C. Azodiisobutyronitrile acted both as initiator of the polymerization and the main foaming agent, and deionized water acted both as a plasticizing agent and an auxiliary foaming agent, which simplified the process and formula. It was found that cavities of two different sizes were formed. The nonuniformity of the foamed material was ascribed to the heterogeneous foaming precursor consisting of a rigid core and a soft shell. Its excellent flame-retard rating of UL-94 V-0 was ascribed to the two nitrogenous layers, which provides a sufficient nitrogen source for non-combustible gases. A relatively high compression strength of 17.7 MPa was partly due to the fiber of corn straw.

## 1. Introduction

Foamed materials made of petroleum products are widely used in packing, transporting, building, and other various fields. These foamed materials are light, heat-insulated, shock-absorbing, sound-absorbing, and easy to be shaped. However, during the production and the using processes of these materials, their flammability and non-degradability cause heavy problems for safety and the environment. When they are used as insulating layers of buildings, these flammable foamed materials have caused many serious fire accidents in many countries. Burning them releases large amounts of toxic gases such as SO_2_, HCl, and NO. In addition, the waste of these materials cannot be easily degraded and many of them are drifting around the ground or floating on the waters, which has formed a new source of pollution.

Many countries give increasing attention to environmental protection. Laws and policies have been put forward to change the situation of the unreasonable utilization of materials. The use of synthetic foamed materials in some fields, for example, the thermal insulation layer of buildings and temporary houses, has been restrained and even prohibited. However, the use of environmentally friendly materials is greatly encouraged. Corn straw is a kind of natural resource with vast amounts every year in many areas of the world. Unfortunately, in some countries, unwanted corn straws, as well as other straws, are randomly burned by farmers or left to rot upon roads, which pollutes the air and leads to traffic accidents. For this, more and more countries give great encouragement to the reasonable use of corn straws, and researchers are paying more attention to this field. Corn straw is very cheap, even free, and biodegradable. More importantly, from the viewpoint of the preparation and application of materials, corn straw contains considerable porous structures at its central part and large amounts of big and strong biofibers at its out-ring part, both of which are beneficial for making light and strong foamed materials. Scientists believe that it is an important development direction to make foamed materials using straw. Many important advances have been made in this field [1,2], but most works have mainly focused on simple mixtures [1,3] of straw with other polymers. Simple grinding or chemical treatment of straw was used in these studies. Some of them used a large amount of adhesives [4], and some potential problems caused by adhesive, for example, the long-term release of formaldehyde, exist in the final products. Furthermore, many of the current foamed materials based on corn straw hold low mechanical strength and cannot fully meet practical needs. There is a realistic need for the development of foamed materials with relatively high strength based on corn straw. An adhesive-less foaming method is a desirable direction, just as that in this study.

Moreover, since foamed materials are frequently used in people’s daily life, flame-retardancy is a necessary requirement for this material. In summary, there are two basic methods to make flame-retardant organic foamed materials. One way is to add flame-retardants—for example, compounds containing halogen, phosphorus, aluminum, or nitrogen—into the matrix. Generally, these compounds exist as separate compositions in the foamed materials. The other way is to design and build some kind of flame-retardant structure in the molecular chains. Furthermore, nowadays, flame-retardants and structures containing halogens are not welcome, since corrosive smoke and toxic combustion products will be released in the using and burning treatment of these materials. Some of these substances may even cause cancer [5,6]. So, nowadays, more attention has been paid to the flame-retardants and structures [7,8] containing B [9], N [10], P [11], Si [10], or other elements [12]. These retardants and structures prevent or restrain combustion through quenching and diluting effects [13], charring, [14] and barriers [15] effects, or through an intumescent [16,17] effect.

In this experiment, a kind of corn straw was ground and then pretreated with chemicals to remove lignins, which are believed to be harmful to the strength improvement of the final products due to their isolation effect to cellulose [18]. A N-containing compound, 1H-imidazole-4-carboxylic acid (C_4_H_4_N_2_O_2_, abbreviated as Im4A in this article), was designedly used for the construction of a flame-retardant structure through an esterification reaction in this experiment. Im4A was acylated using thionyl chloride (SOCl_2_) to improve its esterification reactivity. The chemically pretreated corn straw was esterified by the acylated Im4A. After the esterification, the resulting ester was grafted with allyl chloride and the product was foamed through a heat process in a mold at the presence of water, acrylamide, and azodiisobutyronitrile (AIBN). A simultaneous process of grafting from acrylamide was carried out along with the foaming process. AIBN and water were the main foaming reagent and the auxiliary one, respectively. The structures, morphologies, flammability, and strengths of the intermediates and the final foamed products of the experiment were systematically tested using FT-IR, XRD, SEM, a burning tester, and a universal testing machine.

## 2. Materials and Methods

### 2.1. Materials

Dry corn straws (CStr) were collected from farmland in Yuzhong County of Gansu Province, China. Sodium hydroxide (NaOH), analytically pure, was purchased from Tianjin Baishi Chemical Co., Ltd. (Tianjin, China). Sodium sulfite (Na_2_SO_3_), analytically pure, was purchased from Zhengzhou Danfeng Chemical Co., Ltd. (Zhengzhou, China). Ethanol (C_2_H_5_OH), analytically pure, was purchased from Shanghai Jiumao Trading Company of Pharmaceutical Chemical (Shanghai, China). 1H-imidazole-4-carboxylic acid (C_4_H_4_N_2_O_2_, abbreviated as Im4A in this article), 98%, was purchased from Shanghai Honghao Bio-Pharm Co., Ltd. (Shanghai, China). Thionyl chloride (SOCl_2_, 98%) and Triethylamine ((C_2_H_5_)_3_N, 98%) were purchased from Hangzhou Songye Chemical Co., Ltd. (Hangzhou, China). *N*,*N*-Dimethylformamide (C_3_H_7_NO), 97%, was purchased from Shandong Xiya Chemical Co., Ltd. (Linyi, China). 4-Dimethylaminopyridine (C_7_H_10_N_2_, DMAP), 97%, was purchased from Shandong Jiuchong Chemical Co., Ltd. (Shouguang, China). Magnesium sulfate (MgSO_4_), 99%, was purchased from Nanjing Shengqinhe Chemical Co., Ltd. (Nanjing, China). Allyl chloride (CH_2_=CHCH_2_Cl), 98%, was purchased from Hefei Evergreen Chemical Co., Ltd. (Hefei, China). Azodiisobutyronitrile (AIBN, [(CH_3_)_2_C(CN)]_2_N_2_), 98%, was purchased from GL Biochem Co., Ltd. (Shanghai, China). Acrylamide (C_2_H_3_CONH_2_), 97%, was purchased from Beijing Xinsaiwei Chemical Technology Co., Ltd. (Beijing, China). Homemade deionized water was used.

### 2.2. Instruments

Scanning electron microscopy (SEM), JSM-5600LV (JEOL Ltd., Akishima, Japan); Fourier transformation infrared spectrometer (FT-IR), ENSOR 27 (Bruker, Heidelberg, Germany); X-ray diffraction (XRD), X’ Pert PRO (PANalytical B.V., Almelo, The Netherlands); Flame-retardant performance tester with horizontal and vertical methods, M607 (Qingdao Shanfang Instrument Co., Ltd., Qingdao, China); Universal material testing machine, DY35 (Adamel Lhomargy, Division d’Instruments S.A., Longjumeau, France); Flat vulcanizing press machine, MN (Nanjing Rubber Machinery Factory Co., Ltd., Nanjing, China).

### 2.3. Methods

#### 2.3.1. The Preparation and Characterization of the Flame-Retardant Foaming Material Using Modified Corn Straw

**Figure 1:**
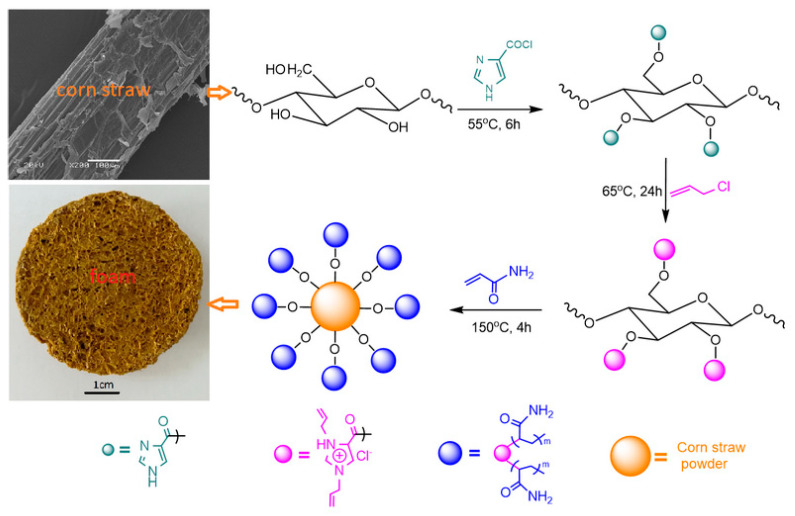
Schematic representation of the flame-retardant foamed material with two nitrogenous layers.

Dry corn straws (CStr) with leaves were collected from farmland in Yuzhong County, Gansu Province of China. Figure 1 schematically depicts the main processes of the experiment, including the esterification of the corn straw, the graft from allyl chloride, and the graft from acrylamide. The pretreatment of corn straw, the acylation of 1H-imidazole-4-carboxylic acid, the foaming process, and the analysis of the materials are not shown in Figure 1. The detailed information of these processes are depicted in the relevant sections below.

#### 2.3.2. The Preparation and Test Processes of the Flame-Retardant Foamed Material

The first step was the pretreatment of the corn straw powders. Corn straw mainly consists of cellulose, semi-cellulose, and lignins. The lignins, as well as small amounts of SiO_2_ and esters on the surface of cellulose, though needed in the growth of plants, are adverse to the direct combination between celluloses or cellulose with the other compositions. So, firstly, the corn straw powders were treated with chemicals to remove lignins, SiO_2_, and esters. Collected corn straws were cut into short pieces of about 5 cm long. After being washed with water and dried in an oven, the corn straws were smashed into fine powders using a grinder with sharp high-speed rotating knives. Then, we sieved the powders through a Tyler screen of 100 meshes and maintained the powders under the screen. The corn straw powder of 20 g was poured into a flask of 500 mL containing a mixed solution of about 200 mL, which was prepared at room temperature by adding 100 mL aqueous NaOH of 2.5 M and 100 mL aqueous Na_2_SO_3_ of 0.4 M. In an oil bath pan, the mixture was heated for 7 h under reflux state. The powder was then filtrated and washed using deionized water until the water became neutral. The powder was then dried in an oven at 60 °C for 2 h. The pretreated corn straw powders were thus obtained. FT-IR and XRD analyses of the powder were carried out to explore the possible changes in structure and composition.

The second step was the esterification of the corn straw powders, of which the lignins, SiO_2_, and esters had been removed in the first step. The main purpose of the esterification was to incorporate a chemical structure, imidazole ring, which contains a N element and was specially designed for the construction of a flame-retardant structure. The main composition of the corn straw powders was now cellulose, which consists of linear 1,4-β-glucans with hydroxy groups at its C-2, C-3, and C-6 positions. Though there are large amounts of hydroxy groups in their long molecular chains, those hydroxy groups involved in intra- or intermolecular hydrogen bonds are hard to be esterified by an acid such as 1H-imidazole-4-carboxylic acid (Im4A). So, before the esterification, Im4A was acylated using thionyl chloride (SOCl_2_) to improve its esterification reactivity. The acylated product was 1H-imidazole-4-carbonyl chloride, abbreviated as Im4ylCl in this article. The pretreated corn straw powders, of which the main composition was cellulose, were then esterified by the 1H-imidazole-4-carbonylchloride (Im4ylCl) and the produced ester was abbreviated as cell-ylIm4.

In the acylation process, Im4A of 5.0 g was added into a flask of 300 mL containing SOCl_2_ of 6.4 g. In this reaction, there was an excess of 20% in the amount of SOCl_2_. The mixture was heated in an oil bath pan to 60 °C and kept at this temperature for 4 h under reflux condensation. After this, the unreacted SOCl_2_ was removed using a rotary evaporator. The acylation product, 1H-imidazole-4-carbonylchloride (Im4ylCl), pale yellow particles, was dried in an oven at 60 °C for 2 h. Then, in the esterification process, the pretreated corn straw powder of 6 g was added into a flask of 500 mL containing 14.4 g of Im4ylCl. N, N-Dimethylformamide (C_3_H_7_N, used as solvent) of 205 mL, triethylamine ((C_2_H_5_)_3_N, used as acid acceptor) of 0.35 mL, and 4-Dimethylaminopyridine (C_7_H_10_N_2_, used as catalyzer) of 0.27 g were subsequently added into the flask and the mixture was heated to 55 °C using an oil bath pan and kept at this temperature for 6 h. The product was washed using deionized water to remove soluble substances by a suction filter until the water became neutral, and then dried in an oven at 60 °C for 2 h. Dry powders of cell-ylIm4 were thus obtained.

In the third step, a kind of long plastic carbon chain consisting of repeated units of -CH_2_CH_2_- was grafted onto the product of the second step, cell-ylIm4. Although there are a large amount of long molecular chains in the cellulose of the corn straw, those chains lack plasticity, which restrained their formability. So, a kind of long plastic chain was prepared on the surface of the cellulose in the third step, the main purpose of which was to endow the cellulose formability. Two monomers, allyl chloride (C_3_H_5_Cl) and acrylamide (C_3_H_5_NO), were sequentially used. Our experiment had tested that allyl chloride is an active and effective monomer in the reaction with cell-ylIm4; acrylamide could bring more N elements, which were expected to act as flame-retardant factor in this experiment, into the outer part of the anticipated long plastic chain.

The graft process can be divided into two stages. In the first stage, allyls were grafted onto cell-ylIm4. That is, each imidazole ring in cell-ylIm4 was grafted with two allyls at its N-1 and N-3 positions. Due to the existence of a polymerization inhibitor in allyl chloride, it was believed that the end of an allyl was not prolonged further by a possible additional polymerization reaction at its double bond position. In this process, cell-ylIm4 of 10.0 g was added into a flask of 500 mL containing 10.4 g of allyl chloride (C_3_H_5_Cl, containing inhibitor), and the mixture was heated to 65 °C and kept at this temperature for 24 h with N_2_ protection. After this, the unreacted allyl chloride was removed using a rotary evaporator. The product was simply marked as cell-ylIm4-2(C_3_H_5_). It is an ionic liquid [19,20], and was used in the next step without washing or other refining.

In the fourth step, the final product of the third step, cell-ylIm4-2(C_3_H_5_), was further grafted from acrylamide, a monomer containing N element. This process can be viewed as the second graft stage; the product, marked as cell-ylIm4-2(C_3_H_5_)-2(C_2_H_3_CONH_2_)m, was foamed in a mold under heating and compression. In one typical formula of this process, which was adjusted systematically, deionized water (used as a plasticizing agent and an auxiliary foaming agent) of 15 mL was added into a beaker of 50 mL. Acrylamide (C_2_H_3_CONH_2_, monomer) of 15 g was added into the beaker. The mixture was stirred until the acrylamide was totally dissolved. Azodiisobutyronitrile ([(CH_3_)_2_C(CN)]_2_N_2_, AIBN, initiator and main foaming agent) of 1 g, which had been ground into fine powder, was added into the beaker and mixed well. Cell-ylIm4-2(C_3_H_5_) of 7.5 g was poured into the beaker and mixed well. A uniform paste was formed and was then poured into a mold with a quadratic cavity of 4 cm wide. The height of the cavity was sufficient for the foaming process and its upper lid was far away from the upper surface of the paste before the foaming process. The position of the upper lip in the mold could be adjusted, which also adjusted the foaming ratio when the origin volume of the paste was constant and less than that of the cavity. We put the mold on a flat sulphuration machine and kept it at 150 °C for 4 h, then a piece of foam was prepared. A polymerization reaction, from which a compound marked as cell-ylIm4-2(C_3_H_5_)-2(C_2_H_3_CONH_2_)m was formed, and a foaming process were carried out simultaneously in this process. The modified corn straw powder has a core/shell structure in which rigid cellulose fiber bundles act as the core and the long soft chains act as the shell.

Tests and analysis of the intermediate products and the final foamed materials were conducted using various methods, including Fourier transform-infrared spectroscopy (FT-IR) characterization, X-ray diffraction (XRD) characterization, scanning electron microscope (SEM) characterization, a mechanical strength test, an apparent density test, and a flammability test. Some of these tests were carried out between two steps with the purpose of testifying the structure of intermediate involved reactions. Detailed processes of these tests are depicted below.

### 2.4. Analysis of the Materials

#### 2.4.1. Fourier Transform-Infrared Spectroscopy (FT-IR) Characterization

These samples were analyzed with Fourier transform-infrared spectroscopy (FT-IR). KBr and corn straw, original or modified, were mixed together and then the mixture was pressured into tablets. The final foamed material was also tested. The spectra of these samples were tested and recorded by a spectrometer, ENSOR 27 (Bruker, Germany), at the range of 4000–400 cm^−1^.

#### 2.4.2. X-ray Diffraction (XRD) Characterization

Structures of the corn straw, original or modified, and the final foamed material, were characterized by an XRD diffractometer, X’ Pert PRO (PANalytical B.V., The Netherlands), in the diffraction angle (2θ) range from 5.0° to 40.0° at a scanning rate of 1.0°/min and a sampling interval of 0.05°, 40 kV, 40 mA, CuK_α_.

#### 2.4.3. Scanning Electron Microscopy (SEM) Analysis

A small amount of the corn straw powders, original or modified, were dispersed with a little drop of alcohol on a small copper disk. To the foamed material, a little piece of it was stuck onto a copper disk using a piece of conductive tape. After being sprayed with gold under a vacuum, these samples were observed with scanning electron microscopy (SEM, JSM-5600LV, JEOL Ltd., Japan).

#### 2.4.4. Apparent Density

The apparent density of the foamed materials was measured according to a Chinese standard, GB/T 6343-2009, which is equal to ISO 845-2006. Three regular cube samples were cut from a piece of foamed material. Their weight were measured using a balance, and their length, width, and height were measured using a vernier caliper. The apparent density of these samples was calculated according to the formula below:

*ρ* = *m*/(*abc*)

in which *m* is the weight of the sample and *a*, *b*, and *c* are the length, width, and height of the sample. Three data were obtained and the average value of them is provided as the apparent density of the material in this paper.

#### 2.4.5. Foaming Ratio

In this study, the foaming ratio was defined as the ratio of the volume of the foamed material to that of the paste before the foaming process. In the experiment, since the foaming process was carried out in a mold with a square cavity, this ratio was also equal to the ratio of the height of the foamed material to that of the paste.

#### 2.4.6. Mechanical Strength Test

The compression strength and the elastic resilience of the foamed material were tested according to the Chinese standard of GB/T 10654-2001, which is equal to ISO 1798-1997. The move speed of the beam on the universal material testing machine (DY35, Adamel Lhomargy, France) used in this test was 2 mm/min, and each value in this article is the average of 5 tests. The sample size for the compression test was 10 mm × 10 mm × 4 mm.

#### 2.4.7. Flammability Test

The flammability of the foamed material was tested according to GB 4609-84, a Chinese standard which is equal to ANSI/UL-94-1985. This test standard is for the vertical burning test. Each value in this article is the average of 5 tests. The sample size for this test was 130 mm × 13 mm × 3 mm. According to this standard, the flammability resistance level of a certain material is sorted from high to low as V-0, V-1, and V-2. Liquid gas was used as burning gas. Ignition duration was 20 s and flame height was about 30 mm.

## 3. Results and Discussion

### 3.1. The First Step: The Pretreatment of Corn Straw

In the first step, corn straw was pretreated using chemicals and comparison analysis was made between the original and the treated corn straw. FT-IR and XRD analyses were used in this step and in the subsequent steps to testify the structure information of the final foamed material and the intermediate products of involved treatment or reactions.

**Figure 2:**
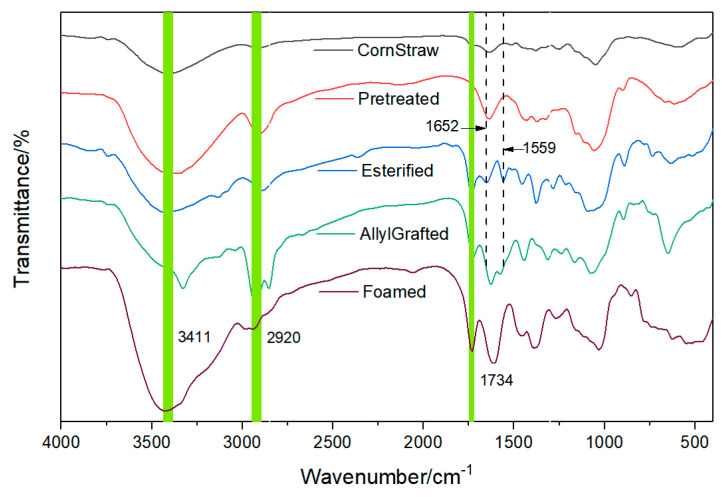
FT-IR spectra of the intermediates and the foamed material.

The FT-IR spectra of the corn straw, before (named CornStraw) and after (named Pretreated) the pretreatment are shown in Figure 2. Before the pretreatment, it can be seen from CornStraw that the broad peak centered at 3411 cm^−1^ is of the stretching vibration of the O-H bond, and 2920 cm^−1^ is of the stretching vibration of the C-H bond. Large areas of the two bands proved the abundance of the two bonds in cellulose, semi-cellulose, and lignins of the corn straw. The small peak centered at 1734 cm^−1^ is of the stretching vibration of the carbonyl group of some esters, which confirms that the existence of esters in the corn straw are in small amount. The peak centered at 1513 cm^−1^ is of the stretching vibration of benzene rings, 1301 cm^−1^ is of the stretching vibration of syingyls, and 1040 cm^−1^ is of the plane bending vibration of guailcyls. The latter three groups come from lignins, which are harmful compositions in the designed reaction of this study and should be removed. After the pretreatment, it can be seen from the Pretreated curve that these three peaks disappeared, which indicate that the lignins were removed. Furthermore, in Pretreated, there is no peak at around 1734 cm^−1^, which is of the stretching vibration of the C=O bond of some esters. This indicates that the esters were also removed by the treatment. After the acylation reaction, peaks appeared at 1734 cm^−1^ in the three spectra in the lower region of Figure 2.

**Figure 3:**
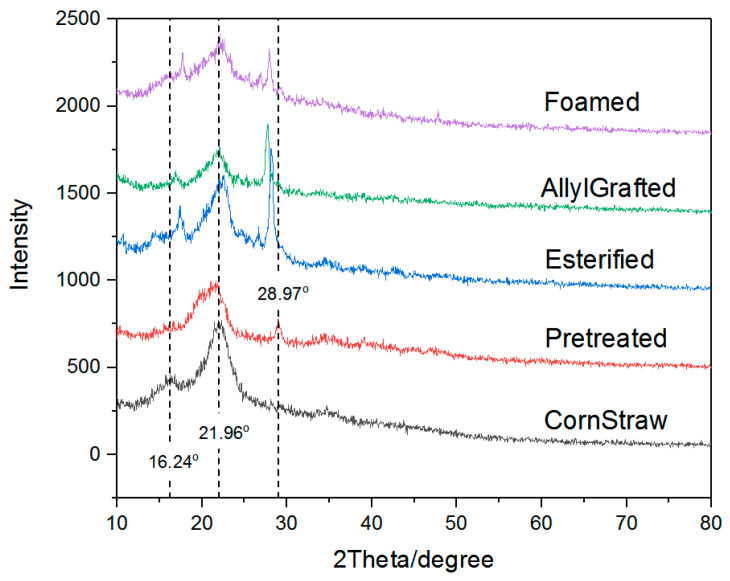
XRD spectra of the intermediates and the foamed material.

Figure 3 shows the XRD spectra of the corn straw, before (CornStraw) and after (Pretreated) the pretreatment. After the pretreatment, it can be seen from the Pretreated curve that a small peak appeared at 2θ = 28.97°, and the peak at 16.24 (1 0 −1) [21] disappeared. Compared with CornStraw, the peak centered at 2θ = 21.96° (0 0 2) was broadened. This indicates that the crystallinity of the corn straw was decreased by the chemical treatment. Research has found that the cellulose is composed of an alternately distributed crystal region and amorphous region, and the crystal region of the cellulose mainly consists of microcrystallines of eight-chain unit cells [22], which is a triclinic lattice cell with two cellulose chain segments running parallel [23] along the fiber axis [24]. Hydrogen bonds are influential forces to the microcrystallines [25] in the cellulose [18]. After the pretreatment, FT-IR analysis proved that lignins were removed from between some parts of the cellulose and semi-cellulose, and it was believed some of the hydrogen bonds were interrupted by OH^−^ used in the pretreatment, which caused the decrease in the crystallinity of the corn straw. As summarized by A. D. French [26], three typical methods based on XRD analyses, namely the Segal peak height method [27], the peak deconvolution method [28] and the Rietveld method [29], have been used in the evaluating of cellulose crystallinity. Due to its easiness and robustness, the Segal peak height method was used in this study.

### 3.2. The Second Step: The Esterification of the Pretreated Corn Straw Using a Nitrogen-Containing Compound

As mentioned above, the main purpose of the esterification of the corn straw was to incorporate a nitrogen-containing structure, imidazole ring, which was designed in this study for the construction of a flame-retardant structure. A nitrogen-containing acid, 1H-imidazole-4-carboxylic acid (C_4_H_4_N_2_O_2_, Im4A), after being acylated using thionyl chloride (SOCl_2_), was used to esterify the corn straw powder (pretreated). Both the acylation and esterification reactions are depicted in Figure 1. The acylated product and the esterified product were abbreviated as Im4ylCl and cell-ylIm4, respectively.

In Figure 2, a comparing analysis of the pretreated corn straw (the Pretreated curve in Figure 2) and the ester (cell-ylIm4, named Esterified in Figure 2) found that, in the Esterified curve, the broad band centered at 3411 cm^−1^, corresponding to the stretching vibration of O-H bonds, was relatively smaller than that in the Pretreated curve, which indicates that some, but not all, of the O-H bonds in the pretreated corn straw were superseded by the esterification reaction. There are three possible positions of the O-H bonds which were not superseded: the ones on the inner parts of the cellulose bundle, the ones on the surface of the bundle but restrained by some factor such as steric hindrance or strong hydrogen bond, or the combination of the two. It is a pity that this study failed to distinguish them. In the Esterified curve, a sharp peak centered at 1740 cm^−1^, which is for the stretching vibration of the C=O bond, and a peak centered at 1215 cm^−1^, which is for the C-O-C bond, appeared, which indicates the formation of esters. The sharp peaks centered at 1652 cm^−1^ and 1559 cm^−1^ are for the stretching vibration of C=C bond and C=N bond in the imidazole rings of the produced esters, respectively.

It can be found from Figure 3 that, after the esterification reaction, in the Esterified curve, compared with the Pretreated curve, two peaks appeared at 2θ = 17.86° and 27.81°, which are believed to belong to the newly formed esters. In addition, compared with Pretreated, the peak centered at about 2θ = 21.96° moved upward to about 22.16° and became narrow, which is ascribed to the spacing decrease of the cellulose. The spacing decrease of the cellulose is believed to be a structure adjustment of the long pyranose chains of the cellulose, which are linked by hydrogen bonds and parallel to one another. The esterification reaction involved some of these hydrogen bonds and changed the spacing between them. The slight downward offset of the peak centered at 28.97° in the Pretreated curve also indicates this kind of influence of the esterification.

### 3.3. The Third Step: The Graft of the Esters Using Allyl Chloride

The pyranose chains of the cellulose of the corn straw lack plasticity, which restrained its formability. So, a kind of long plastic chain was grafted onto the imidazole rings on the pyranose chains using allyl chloride (CH_2_=CHCH_2_Cl) in the third step, and then the product was further grafted using acrylamide (C_2_H_3_CONH_2_) in the following step. Allyls are easily grafted onto the imidazole rings, but the chains formed by the graft of them are at risk of flammability. So, acrylamide was used to construct chains at the outer part of the product with dense N element, which was designed to act as a flame-retardant.

A comparing FT-IR analysis of the ester, before (the Esterified curve, cell-ylIm4) and after being grafted (the AllylGrafted curve, cell-ylIm4-2(C_3_H_5_) using allyl chloride, is shown in Figure 2. The peak centered at 3434 cm^−1^ in the AllylGrafted curve is for the stretching vibration of the remaining O-H bond, which indicates that the esterification reaction in the former step did not supersede all the O-H bonds on the pyranose rings of the corn straw. The sharp shape of the peak indicates the remaining O-H bonds in the pyranoses rings are quite sensitive to Infrared radiation, which seemed to be a result of the grafting reaction of allyl chloride. Furthermore, the peaks centered at 2924.42 cm^−1^ and 853.29 cm^−1^, which are quite strong, are for the stretching vibration of the C-H bonds in -CH_3_ group and -CH_2_- group, respectively. The peak centered at 1703.59 cm^−1^ is for the stretching vibration of C=O bond. The peaks at 1627.52 cm^−1^ and 1573.59 cm^−1^ are for the stretching vibration of the C=C bonds of the allyls and C=N bonds in the imidazole rings, respectively. The peak at 1078.68 cm^−1^ is for the bending vibration of the C-H bond. The peak at 935.44 cm^−1^ is for the plane bending vibration of the C-H bond in the CH_2_=CH- group, which indicates that the -CH_2_CH=CH_2_ group has been grafted onto the cell-ylIm4. The -(CH_2_CH=CH_2_)n was linked at the N-1 and N-3 positions of the imidazole ring and the final product was marked as cell-ylIm4-2(C_3_H_5_).

Figure 3 shows the XRD spectra of the esterified corn straw, before (cell-ylIm4, the Esterified curve) and after (cell-ylIm4-2(C_3_H_5_), the AllylGrafted curve) grafting with allyl chloride. It can be seen from Figure 3 that the two spectra are almost the same, which indicates that the grafting by allyl chloride did not change the crystalline structure of the esterified corn straw.

### 3.4. The Fourth Step: The Foaming Process Accompanying a Grafting through Acrylamide

In the fourth step, the product of the third step, cell-ylIm4-2(C_3_H_5_), was further grafted through acrylamide. The product was marked as cell-ylIm4-2(C_3_H_5_)-2(C_2_H_4_CONH_2_)m. Using the grafting method described above, acrylamide, a common nitrogen-containing monomer, was used to build a layer with dense N element, which was believed to be a flame-retardant structure, on the surface of the modified corn straw powders. The grafting reaction and the foaming process were simultaneously carried out in a mold under 150 °C. In this step, two chemicals acting as compound functions effectively simplified the formula. For example, azodiisobutyronitrile (AIBN) was used both as the initiator and the main foaming agent, and deionized water was used both as the plasticizing agent and the associate foaming agent. It was realized through a systematical adjusting to the formulation.

A comparing FT-IR analysis of cell-ylIm4-2(C_3_H_5_) (the AllylGrafted curve in Figure 2), which was obtained by the grafting from allyl chloride, and the final foaming material (the Foamed curve in Figure 2), which came from the former mentioned grafting from acrylamide, found that, in Foamed, the broad band at 3421.70 cm^−1^ is for the stretching vibration of the N-H bond of the -NH_2_ group, which came from acrylamide, and the remaining O-H bond on the pyranose rings of the corn straw after the esterification reaction. It was found that after the grafting from acrylamide the peak centered at 935.44 cm^−1^, which is of the plane bending vibration of the C-H bond in the CH_2_=CH- group of propenyl, disappeared. This indicates that acrylamide monomer had been grafted onto cell-ylIm4-2(C_3_H_5_).

**Figure 4:**
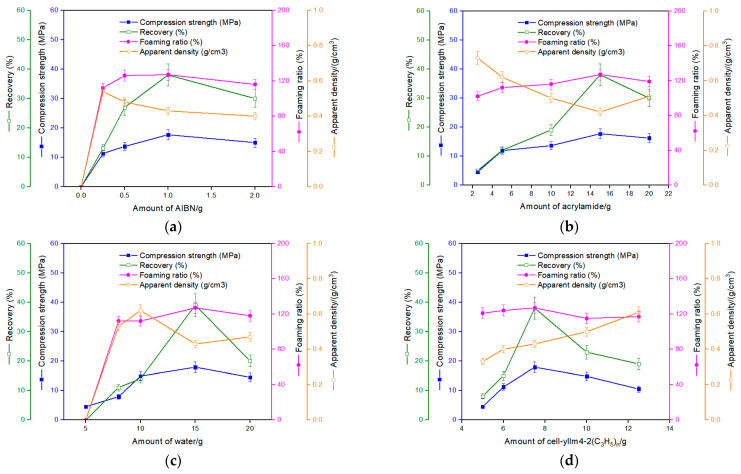
Influence of the amounts of (**a**) AIBN, (**b**) acrylamide, (**c**) water, and (**d**) cell-ylIm4-2(C_3_H_5_) on the properties of the foamed material. In (**a**), *w*_water_ = *w*_acrylamide_ = 15.0 g and *w*_cell-ylIm4-2(C3H5)_ = 7.5 g; in (**b**), *w*_water_ = 15.0 g, *w*_AIBN_ = 1.0 g, and *w*_cell-ylIm4-2(C3H5)_ = 7.5 g; in (**c**), *w*_acrylamide_ = 15.0 g, *w*_AIBN_ = 1.0 g, and *w*_cell-ylIm4-2(C3H5)_ = 7.5 g; and in (**d**), *w*_water_ = *w*_acrylamide_ = 15.0 g and *w*_AIBN_ = 1.0 g.

The formulation of the foamed material was systematically adjusted. The foaming process used water, acrylamide, cell-ylIm4-2(C_3_H_5_), and AIBN as raw materials. Keeping the amounts of three compositions of them constant, the influence of the amount of the other one on the properties of the foamed material was investigated, and the results are shown in Figure 4.

**Figure 5:**
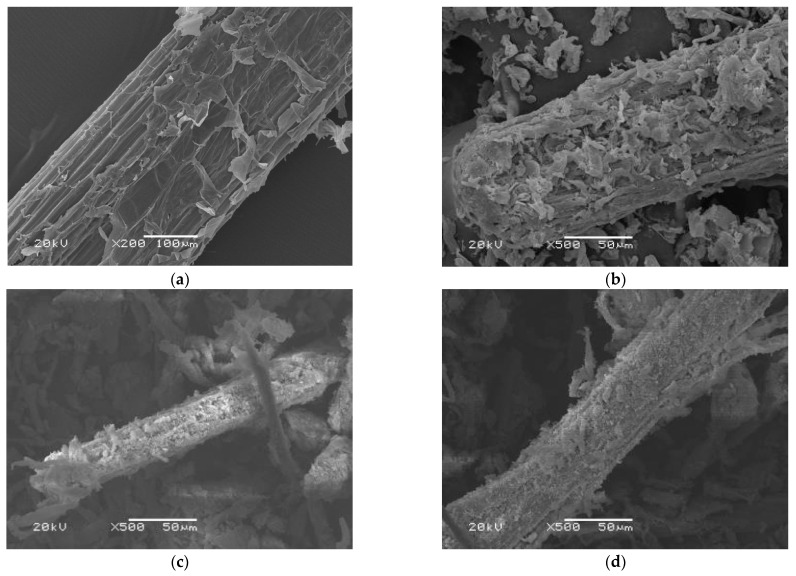

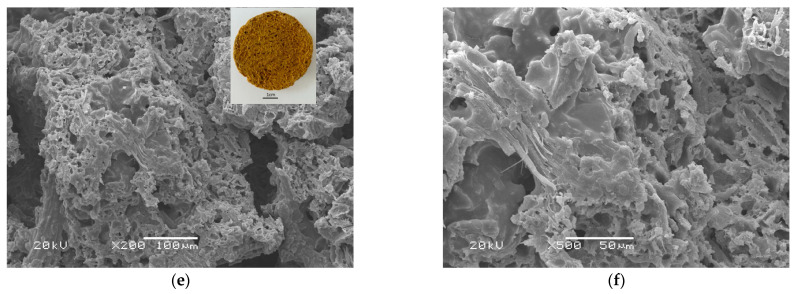
SEM images of corn straw of (**a**) the original, (**b**) the pretreated, (**c**) the esterified, and (**d**) the allyl-grafted. Sizes of the cavities in the foamed material (**e**,**f**) are in two levels.

Figure 4a shows the manifest influence of the amount of AIBN on the properties of the foamed material. AIBN was the main foaming reagent. It can be seen from Figure 4a that the compression strength, recovery, and foaming ratio increased first and then decreased with the increase in AIBN. Since AIBN also acted as initiator for the grafting reaction of acrylamide, the absence of it led to a failure in molding, which indicates that the molding process obviously relayed on the expected long chain formed by acrylamide. It was believed that the amount of AIBN had influence on the foaming extent as well as the chain growth extent, and the latter increased with the increase in AIBN, the initiator. The appropriate amount of AIBN formed small and uniform foaming cavities (Figure 5). However, when AIBN exceeded 1 g, the compression strength and recovery decreased, which was ascribed to the bigger cavities caused by excessive AIBN. The drop in apparent density also supported this viewpoint. A foaming ratio of about 112~127% was obtained, which showed a reverse trend compared to that of the apparent density. It seemed that the foaming ratio was controlled more by the strong adhesion, which came from the remaining intermolecular hydrogen bonds in the fiber bundles of the modified corn straw, than by the amount of AIBN, the foaming agent in this experiment. As can be seen from Figure 5, the fiber bundles kept their wholeness and had not been totally separated by the modifying process. It also indicates that AIBN could not enter the intersite of the fiber bundle of the corn straw.

As shown in Figure 4a, the best properties of the foamed material were obtained when AIBN was 1.0 g. So, in the subsequent trials, AIBN was kept constant at this amount to investigate the influence of the other compositions. Figure 4b–d sequentially show the influence of the amounts of acrylamide, water, and cell-4ylIm4-2(C_3_H_5_). Acrylamide or water of 15.0 g and cell-4ylIm4-2(C_3_H_5_) of 7.5 g were believed to be the appropriate amounts by a comprehensive consideration of the data in Figure 4b–d. The foamed material made in this experiment felt far harder than the commonly used foamed materials of PE, PVC, PS, and PU. Through the optimized formula, a compression strength of about 17.7 MPa was obtained at a relatively little strain (about 15% of the original height of the test sample), which indicates that the foamed material as prepared holds relatively higher anti-pressure ability and can provide a large stress by a relatively little strain, which is favorable to applications for buffer conditions, such as packing material for some heavy goods.

What needs to be specially noted is that water, an abundant and cheap natural resource, after being deionized, was used in this experiment both as solvent and auxiliary foaming agent. It can be seen from Figure 4c, when water was equal or less than 10.0 g and the compression strength was low, which was imputed to insufficient dissolution of acrylamide, that a short molecular chain formed at the surface of the modified corn straw powders. However, water of 20.0 g made the foamed material less strong when pressured, which was ascribed to the cavities of large size caused by slow release of the excessive water. In this study, a suitable amount of water was largely equal to that of acrylamide in weight.

### 3.5. Observation of the Intermediate Products and the Foamed Material

Figure 5 shows the SEM images of the corn straw of the original (Figure 5a), the pretreated (Figure 5b), the esterified (Figure 5c), the allyl-grafted (Figure 5d), and the foamed material (Figure 5e,f). As shown in Figure 5a, the original corn straw powders were mainly composed of fiber bundles with diameters of 20~100 μm, and some little pieces randomly adhered onto or scattered around these fiber bundles. After the pretreatment, and then the esterification and the graft through allyl, the bundles became loose to some extent but still kept their rodlike profiles, and the little pieces became more (Figure 5b–d).

After a simultaneous process of grafting (through acrylamide) and foaming under 150 °C, a hard foam was obtained, SEM images of which are shown in Figure 5e,f. The foam cavities were not uniform both in size and shape. There were two levels of foam cavities: the smaller ones were in several microns and were generally unconnected, and the larger ones were in about ten to one hundred microns and connected. It was believed the nonuniformity in the size and shape of the foam cavities was caused by the heterogeneous precursor, which in the foaming process was a paste composed of rigid fiber bundles (as core) and long soft chains (as shell). A little picture in Figure 5e shows some big holes in the surface of the foamed material, which are passageways of the gas released during the foaming process.

### 3.6. Flammability Test Result

A vertical burning test of the foamed material obtained by the optimized formula showed that its glowing time and after-glow time were 6 s and 0 s, respectively; a horizontal burning test showed that its glowing time and after-glow time were 2 s and 0 s, respectively. Its flame-retardant rating was UL-94 V-0, which indicates that the foamed material held an excellent flame-retardancy. We ascribed this excellent flame-retardancy to the double nitrogenous layers of the modified corn straw powders. The inner thin nitrogenous layer consisted of imidazole rings, which came from an esterification reaction of the corn powder with 1H-imidazole-4-carbonyl chloride, and the outer thick nitrogenous layer was prepared by grafting acrylamide onto an intermediate layer through a free-radical polymerization. A black char crust was soon formed on the surface during the burning test and was believed to effectively prevent heat transfer. Horacek et al. [30] believe that a char crust formed by burning, which has a closed and regular cell structure filled with a heavy gas with low thermal conductivity, such as carbon dioxide, would hold high efficiency of flame-retardancy. We believe that oxides of nitrogen [31] released during the burning test, which were non-combustible [32] and had bad thermal conductivity [17], acted a similar function as that of carbon dioxide, and the char crust acted as a preventive layer to heat transfer. Based on this mechanism, the foamed material holds good flame-retardancy.

## 4. Conclusions

A foamed material with an excellent flame-retardant rating of V-0 was prepared from a kind of modified corn straw powder. There was an intermediate of ionic liquid in the modifying process.

- The modified corn straw powder had a core/shell structure, in which rigid cellulose fiber bundles acted as the core and the long soft chains acted as the shell. The long soft chains contained two nitrogenous layers. The inner thin nitrogenous layer consisted of imidazole rings, which came from an esterification reaction of the corn powder with 1H-imidazole-4-carbonyl chloride, and the outer thick nitrogenous layer was prepared by grafting acrylamide onto an intermediate layer through a free-radical polymerization. The excellent flame-retardancy was ascribed to the two nitrogenous layers, which provided a sufficient nitrogen source for non-combustible gases. These gases, along with the char crust, effectively prevented the heat transfer and retarded the flame.
- Azodiisobutyronitrile (AIBN) acted as an initiator and the main foaming agent, and deionized water as a plasticizing agent and an auxiliary foaming agent. This effectively simplified the formula.
- Two levels of foam cavities, one of several microns and generally unconnected, and the other of ten to one hundred microns and connected, were formed. It is believed the nonuniformity was caused by the heterogeneous precursor, which was composed of rigid cores of fiber bundles and soft shells of long carbon chains.
- The strength of the final foamed material was relatively high, which was favorable to applications at some conditions with heavy loads. It was believed the high strength partly came from maintaining the fiber form of the corn straw. In addition, a large part of the crystal region of the cellulose remained after the modification reactions and the foaming process, which was also helpful for obtaining foamed material with high strength.

## Data Availability

Not applicable.
